# The Clinical Approach to Interstitial Lung Disease in Childhood: A Narrative Review Article

**DOI:** 10.3390/children11080904

**Published:** 2024-07-26

**Authors:** Simona Drobňaková, Veronika Vargová, László Barkai

**Affiliations:** 1 Department of Paediatrics and Adolescent Medicine, Faculty of Medicine, Pavol Jozef Šafárik University, 040 01 Kosice, Slovakia; veronika.vargova@upjs.sk (V.V.); or barkai.laszlo@uni-obuda.hu (L.B.); 2 Physiological Controls Research Center, University Research and Innovation Center, Óbuda University, 1034 Budapest, Hungary

**Keywords:** interstitial lung disease, children, treatment

## Abstract

Interstitial lung disease (ILD) comprises a group of respiratory diseases affecting the interstitium of the lungs, which occur when a lung injury triggers an abnormal healing response, and an inflammatory process leads to altered diffusion and restrictive respiratory dysfunction. The term “interstitial” may be misleading, as other components of the lungs are usually also involved (epithelium, airways, endothelium, and so on). Pediatric conditions (childhood interstitial lung disease, chILD) are different from adult forms, as growing and developing lungs are affected and more diverse and less prevalent diseases are seen in childhood. Diffuse parenchymal lung disease (DPLD) and diffuse lung disease (DLD) can be used interchangeably with ILD. Known etiologies of chILD include chronic infections, bronchopulmonary dysplasia, aspiration, genetic mutations leading to surfactant dysfunction, and hypersensitivity pneumonitis due to drugs or environmental exposures. Many forms are seen in disorders with pulmonary involvement (connective tissue disorders, storage diseases, malignancies, and so on), but several conditions have unknown origins (desquamative pneumonitis, pulmonary interstitial glycogenosis, neuroendocrine cell hyperplasia in infancy, and so on). Currently, there is no consensus on pediatric classification; however, age grouping is proposed as some specific forms are more prevalent in infancy (developmental and growth abnormalities, surfactant dysfunction mutations, etc.) and others are usually seen in older cohorts (disorders in normal or immunocompromised hosts, systemic diseases, etc.). Clinical manifestations vary from mild nonspecific symptoms (recurrent respiratory infections, exercise intolerance, failure to thrive, dry cough, etc.) to a severe clinical picture (respiratory distress) and presentation related to the child’s age. The diagnostic approach relies on imaging techniques (CT), but further investigations including genetic tests, BAL, and lung biopsy (VATS) are needed in uncertain cases. Pharmacological treatment is mostly empiric and based on anti-inflammatory and immunomodulatory drugs. Lung transplantation for selected cases in a pediatric transplantation center could be an option; however, limited data and evidence are available regarding long-term survival. International collaboration is warranted to understand chILD entities better and improve the outcomes of these patients.

## 1. Introduction

Interstitial lung disease (ILD) comprises a group of respiratory diseases affecting the interstitium of the lungs, which occur when a lung injury triggers an abnormal healing response, and an inflammatory process leads to altered diffusion and restrictive respiratory dysfunction. The term “interstitial” may be misleading, as other components of the lungs are usually also involved (epithelium, airways, endothelium, and so on) [1]. Pediatric conditions (childhood interstitial lung disease; chILD) are different from adult forms, as growing and developing lungs are affected and as more diverse and less prevalent diseases are seen in childhood. Diffuse parenchymal lung disease (DPLD) and diffuse lung disease (DLD) can be used interchangeably with ILD [2,3].

**Figure 1:**
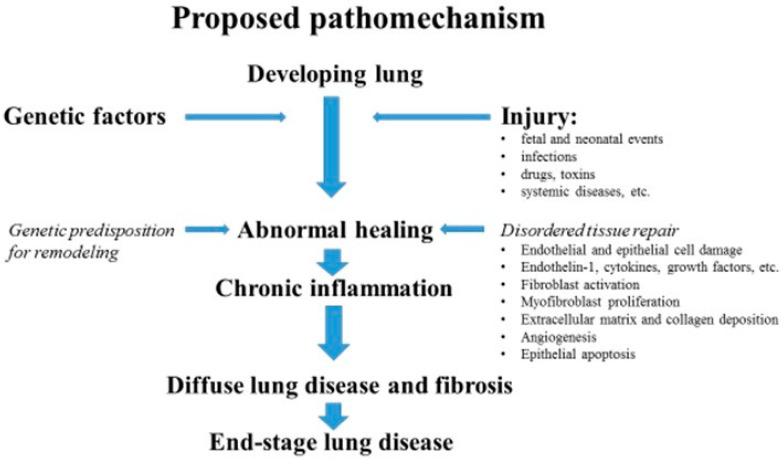
Proposed pathomechanism of end-stage lung disease in children (modified according to Clement, Eber, 2008) [6].

Recent years have seen a growing interest in creating a classification for pediatric diffuse lung disease, including interstitial disease. Such a classification would be instrumental in establishing a structured system for the differential diagnosis process, allowing for the recognition of nuances between adult and pediatric lung disease [4,5,6]. Pathophysiology is more complex in children, as damage occurs during lung growth and differentiation. After repeated injuries to the alveolar epithelial cells and capillary endothelium, an abnormal healing process leads to chronic inflammation, which responds to injury with alveolar–capillary wall thickness, blockade, and, in the end, restrictive respiratory dysfunction. The transformation of epithelial cells can lead to an increased production of collagen and other extracellular matrix components, potentially contributing to the development of progressive fibrosis [5,7]. A schematic representation of the proposed mechanism of diffuse alveolar damage and fibrosis in developing lungs aims to illustrate the complex processes involved in developing lung damage and scarring. Figure 1 provides a visual explanation of how diffuse alveolar damage leads to fibrosis, offering insights into the pathophysiology of these conditions.

Childhood interstitial lung disease (chILD) is rare, with a prevalence ranging from 0.13 per 100,000 children under 17 years of age to 16.2 per 100,000 children under 15 years of age, compared to a prevalence of 60–80 per 100,000 in adults [8].

The aim of this study is to present a review of interstitial lung disease among the pediatric population. Through improving our understanding of these complex conditions, we can make timely and accurate diagnoses, resulting in better patient care.

## 2. Etiology

chILD presents a spectrum of severity, ranging from mild to life-threatening. Notable distinctions between ILD in pediatric and adult populations encompass the criteria for defining the condition, its pathophysiology, etiology, classification, and therapeutic approaches. The histologic classification of interstitial lung disease (ILD) holds varying clinical significance between pediatric and adult populations. While certain etiologies of ILD in children and adults may overlap, the classification of childhood ILD encompasses a diverse range of distinct categories [9,10].

Known etiologies of chILD include chronic infections, bronchopulmonary dysplasia, aspiration, genetic mutations leading to surfactant dysfunction, and hypersensitivity pneumonitis due to drugs or environmental exposures [11]. Many forms are seen in disorders with pulmonary involvement (connective tissue disorders, storage diseases, malignancies, and so on), but several conditions have unknown origins (desquamative pneumonitis, pulmonary interstitial glycogenosis, neuroendocrine cell hyperplasia in infancy, and so on) [12,13].

The chILD Research Cooperative and the European Union–chILD collaboration network have delineated chILD into three distinct categories: immediately after birth, first 2 years of life, and from 2 to 16 years of age. Common chILD manifestations among children under the age of 2 encompass developmental disorders, genetic surfactant disorders, neuroendocrine cell hyperplasia of infancy (NEHI), and pulmonary interstitial glycogenosis. Instances of chILD across all age groups may be attributed to diverse medical conditions including infections, other pulmonary afflictions, immunological aberrations, and environmental exposure to dust or mold [14,15].

Air pollution represents a significant global challenge as harmful pollutants are emitted into the atmosphere by various sources, including vehicular exhaust, industrial activities, biomass fuel combustion, and indoor contaminants. Notably, air pollution has been correlated with diminished lung function, exacerbations of idiopathic pulmonary fibrosis (IPF), heightened prevalence of hypersensitivity pneumonitis (HP), and the manifestation of pulmonary fibrosis in healthy adults and children [16].

The categorization of chILD by etiology is shown in Table 1.

## 3. Clinical Presentation and Classification

The disease can present with a wide range of clinical symptoms, from mild and nonspecific to very severe. This variability makes it challenging for both physicians and patients to pinpoint the exact onset of the illness. The insidious nature of the disease’s onset adds to the difficulty in recognizing when the condition first began [17].

In neonates with surfactant dysfunction mutations, symptoms are more dramatic with progressive respiratory distress syndrome [18]. Tachypnea occurs in 75–93% of patients. Younger infants manifest dyspnea, failure to thrive, sweating with feeding, gastroesophageal reflux, pulmonary hypertension, and chest wall deformities. Cyanosis, a bluish discoloration of the skin or mucous membranes, may become apparent in some individuals during feeding or while at rest. A dry and non-productive cough is commonly present and can be the only symptom of ILD, even in neonates [19]. The presence of hemoptysis can be indicative of an underlying vasculitic process, which involves inflammation of the blood vessels, or pulmonary hemorrhage syndrome [20]. In older children, symptoms such as chest pain, exercise intolerance, and impaired growth may manifest. Furthermore, advanced stages of the condition may present with digital clubbing. Desaturation can occur during periods of sleep, feeding, or physical exertion. Signs indicative of hyperinflation, including an increased chest diameter or the presence of a palpable liver and spleen, may be observable. In the case of infection, fever presents as a general nonspecific sign. Pulmonary auscultation may be normal or with adventitious sounds such as a crackle coupled with wheezing (40%) [21].

In 2013, Kurland et al. introduced the term “chILD syndrome” to identify a phenotype that required further diagnostic evaluation. According to this term, we diagnose this syndrome when a child (<2 years old) with diffuse lung disease (DLD) has common causes of DLD excluded as the primary diagnosis and has at least three of the following four criteria: “(1) respiratory symptoms (cough, rapid breathing and/or difficulty breathing, and exercise intolerance), (2) respiratory signs (resting tachypnea, adventitious sounds, retractions, digital clubbing, failure to thrive, and respiratory failure), (3) hypoxemia, and (4) diffuse abnormalities on chest radiography or high-resolution computed tomography (HRCT)” [22].

At present, there is no consensus on pediatric classification. However, certain forms of the condition are more commonly found in infants (such as developmental and growth abnormalities, surfactant dysfunction mutations, and so on), while others are usually seen in older cohorts (disorders in normal or immunocompromised hosts, systemic diseases, and so on) [23,24].

Based on the official American Thoracic Society (ATS) clinical guidelines released in 2013, a chILD classification scheme has been assessed (Table 2). Adult classifications are not useful in pediatrics. This clinicopathologic classification comes from the chILD Research Network and should be used routinely (Table 2) [22]. The chILDRN is categorized into two groups: “Disorders More Prevalent in Infancy” and “Disorders not Specific to Infancy”.

## 4. Diagnostic Approach

Diagnosing chILD requires physicians to maintain a high degree of suspicion due to the diverse and often subtle nature of respiratory symptoms observed in infants and children. Clinicians need to remain vigilant as these symptoms may not be immediately noticeable, potentially leading to a misinterpretation of the condition [25].

A comprehensive prenatal history, neonatal clinical course, and detailed family history are imperative, as certain presentations of chILD may be of genetic origin and could manifest as premature neonatal mortality, unexplained childhood respiratory ailments, or adult interstitial lung disease (ILD). Vital signs, especially pulse oximetry, are used to assess the efficiency of oxygen delivery by hemoglobin. Sequential blood analyses are employed to quantify oxygen and carbon dioxide levels present within the bloodstream [26].

Based on chILD-EU collaboration (The European Research Collaboration for Children’s Interstitial Lung Disease) recommendations [15], Table 3 provides a differential diagnostic aid using clinical, radiological, and histological findings and possible genetic causes of interstitial lung disease in childhood.

Blood and other tissue samples are tested to investigate known genetic disorders or to discover newly associated genes or variants, immune functions, autoantibodies, and environmental organic dust exposures.

The diagnosis of a disorder related to the surfactant protein genes is typically confirmed through DNA analysis, which allows for the identification of the specific gene and provides valuable information on prognosis. Various certified diagnostic laboratories offer next-generation sequencing panels encompassing these genes, as well as others that may present with overlapping characteristics. Deletions and duplications, specifically occurring within the *SFTPB*, *ABCA3*, and *NKX2-1* genes, are documented. Consequently, the assay must exhibit sensitivity to such genetic alterations. Barriers to genetic testing may arise from factors such as the potentially high cost of these studies, which might not be covered by insurance, and the extended turnaround time for reporting results, especially when dealing with a critically ill child [27,28].

Pathogenic variants associated with chILD are inherited due to monogenic diseases in up to 20–30% of cases. The production of functional surfactant involves four surfactant proteins (SPs)—namely, A, B, C, and D—in addition to supplementary proteins. Among these supplementary proteins are ABCA3, a constituent of the ATP-binding cassette protein family, and TTF-1, which stands for thyroid transcription factor 1 [28,29,30,31,32]. An SP-B mutation is generally inherited in an autosomal recessive manner, and its most common mutation is c.397delCinsGAA [33]. An SP-C mutation, characterized by variable effects on lung disease and age of onset, manifests through inheritance in an autosomal dominant pattern or via de novo occurrence. Surfactant proteins, particularly SP-B and SP-C, serve the critical function of averting alveolar collapse at the cessation of expiration through the reduction in surface tension at the air–water interface within the lung alveoli. *NKX2-1* mutations, in addition to lung affection, may lead to neurologic symptoms (chorea, ataxia, hypotonia, and developmental delay) and hypothyroidism. Mutations in FARSA and FARSB may present with extrapulmonary involvement such as cerebral aneurysms and ARS among other things, such as sensorial defects [34]. Mutations in GMCSFR typically manifest in infants and young children, presenting symptoms such as dyspnea, cough, and alveolar proteinosis. These manifestations may result from loss-of-function mutations or deletions affecting both alleles within the *CSF2RA* and *CSF2RB* genes [35]. The synthesis, secretion, and degradation of these proteins are meticulously regulated. The transcription of surfactant genes is reliant on the thyroid transcription factor *NKX2-1*, while the ABCA3 protein governs its intracellular storage and transport. Additionally, the processing and degradation of the surfactant by macrophages are subject to the influence of granulocyte–macrophage colony-stimulating factor (GM-CSF). Any variations in these genes and immune regulation may contribute to surfactant dysfunction [36,37].

Mutations within the surfactant protein (SP) genes play a causative role in the familial variant. Additional genetic variations are delineated in Table 4, providing a comprehensive overview of the distinct genetic forms identified [38].

As per the ATS guidelines, it is strongly recommended to conduct echocardiography to rule out structural cardiovascular disease and assess pulmonary hypertension. Conditions such as left-sided heart pathology or other forms of cardiac involvement within the context of a general illness (pericarditis and associated heart malformations) have the potential to resemble ILD [22].

Pulmonary function tests play a pivotal role in precisely defining the extent of restrictive lung disease and monitoring patient response to treatment. Within the context of ILD, pulmonary function abnormalities can manifest as restrictive ventilator deficits; however, an obstructive pattern characterized by reduced lung volume is also observed in some cases [39].

Chest radiographs (CXRs) serve as the initial imaging modality for assessing pulmonary conditions, including chILD. While CXRs lack specificity and sensitivity, their cost-effectiveness, ease of use, and wide availability render them advantageous. High-resolution computed tomography (HRCT) represents the gold standard for characterizing disease involvement, distinguishing between interstitial, airspace-related, and airway-related presentations. Furthermore, HRCT facilitates the visualization of the parenchymal structure down to the level of the secondary pulmonary lobule. Some children find it difficult to lie still—either because of their age or their medical condition—so we might suggest they are sedated for the CT scan. Some patients with chILD have typical CT patterns, while a classical cobblestone appearance is found in pulmonary alveolar proteinosis [40]. NEHI shows characteristic CT findings with ground-glass opacification and mosaic attenuation, likely due to trapped air [41]. In children with surfactant protein disorders, HRCT demonstrates granular opacities with a thickened interstitium. Later, it shows progressive fibroblastic changes [42].

Multidetector CT (MDCT) is a noninvasive technique for evaluating tracheal anatomy and pathology. In 2023, a retrospective study conducted by Miraftabi et al. examined the distinctive multidetector computed tomography (MDCT) patterns in a cohort of 90 consecutive children diagnosed with chILD through biopsy confirmation. Compared to adults, who have a specific MDCT pattern, this technique could be considered highly compatible with lung histology [43].

Bronchoscopy with bronchoalveolar lavage (BAL) provides helpful information related to cytological and microbiological analysis, bleeding, or aspiration, but usually does not indicate an exact diagnosis. It is considered to be relatively safe, easily performed, and widely available and is one of the diagnostic processes for pulmonary alveolar proteinosis [44].

Obtaining a lung biopsy is often the final step in the process of determining a diagnosis, ensuring that an accurate and definitive conclusion can be reached [45]. Conventional surgical thoracotomy is capable of establishing a precise diagnosis and providing essential guidance for subsequent clinical management and therapeutic interventions [46]. It is advisable to consider this approach for neonates and infants presenting with “chILD syndrome” when conventional diagnostic investigations have failed to identify a specific underlying disease. However, it is imperative to involve a multidisciplinary team to fully leverage the diagnostic benefits. Video-assisted thoracoscopy (VATS) is the recommended approach for obtaining a tissue sample when congenital interstitial lung disease (chILD) is suspected. This method is the preferred choice due to its associated reduction in post-intervention complications, shorter recovery period, and lower incidence of patient discomfort [46,47]. An evaluation of possible systemic disease may also be necessary [46].

## 5. Therapy

At present, there exists no targeted therapeutic regimen for pediatric interstitial and diffuse lung disease. Pharmacological treatment has shown limited evidence of a beneficial effect and is mostly empiric [48,49]. Each child undergoing treatment for chILD receives a personalized medical care plan tailored to the specific underlying cause of the disease. All treatment protocols are designed to proactively prevent disease advancement and active illnesses, alleviate symptoms, and optimize growth and development [48].

The most prevalent pharmacological treatments for patients with chILD are corticosteroids, hydroxychloroquine, and azithromycin. According to European protocols, in 2015, Bush et al. recommended using oral prednisolone and intravenous pulse methylprednisolone before other therapies, with an expected patient response rate of 7–28 days (Table 5) [15]. The adverse effects of systemic corticosteroids may impact various bodily systems, including adrenal and growth suppression, heightened susceptibility to infections, reactivation of latent infections (e.g., tuberculosis), reduced responsiveness to vaccinations, and diminished bone mineral density leading to osteoporosis. Vigilant monitoring of these repercussions by a specialist is imperative [50].

Hydroxychloroquine, known as a disease-modifying antirheumatic drug (DMARD), has been successfully used in numerous instances with surfactant protein C deficiency [51,52,53,54] and has had variable responses in cases of *ABCA3* mutations [8,55,56,57]. Based on the European protocol, it represents a second treatment (with no preference over azithromycin) or a first-line treatment in cases of mild stable chILD. In 2022, Griese et al. performed two trials of hydroxychloroquine in childhood interstitial lung disease for the first time. The results for key endpoints showed no differences between differ between the HCQ and placebo treatment periods [58].

Azithromycin, with its microbicidal and immunomodulatory properties, can suppress hyperimmunity and inflammation. It is commonly utilized in conjunction with corticosteroids and/or hydroxychloroquine [48]. It is important to remember that limited evidence for the use of macrolides has been obtained in chILD, and their role remains marginal and empirical. The potential for microbial resistance to azithromycin must be duly acknowledged and carefully assessed [59].

Supportive measures, such as the administration of oxygen therapy, either continuously or during sleep, may be required to alleviate symptoms and mitigate the progression of pulmonary hypertension (PAH) and cor pulmonale associated with alveolar hypoxia. Children experiencing severe respiratory failure may derive benefit from either invasive or noninvasive ventilation [60]. In cases where pulmonary arterial hypertension (PAH) is present, it is advisable to consider the use of sildenafil in conjunction with anticoagulant therapy, especially if PAH is associated with chronic thromboembolic disease. General recommendations also include nutritional support; treatment with bronchodilators, inhaled steroids, or both; and proton pump inhibitor therapy. It is crucial to manage comorbid conditions such as atopic phenotypes, sleep apnea, dysphagia, and gastroesophageal reflux (GER)-associated chronic aspiration. Additionally, it is important to avoid close contact with individuals who have symptomatic respiratory tract infections, especially during times of elevated respiratory virus activity [61,62]. In instances of pulmonary alveolar proteinosis (PAP), the therapeutic application of whole-lung lavage may be considered as a stabilizing intervention [63].

Lung transplant (LuTx) is an available treatment option for children and infants in severe cases. It is considered after all other therapeutic options have been exhausted, if the potential benefits of the surgery outweigh the risks. However, the post-transplant survival rate shows variation based on the specific disease phenotype and the presence of comorbidities [64]. In 2023, Carlens et al. analyzed 97 patients who underwent LuTx with excellent outcomes. At the time of reporting, 89% of the patients were alive after a median time of 4.65 years post-LuTx for chILD [65]. Data and evidence are currently limited.

### New Therapeutic Options

In the context of children and adolescents affected by fibrosing ILD, ongoing investigations are being conducted to broaden the application of these therapeutic agents. Two drugs with pleiotropic antifibrotic effects (pirfenidone and nintedanib) are licensed treatments in adults [66]. InPedILD is the first international placebo-controlled trial of nintedanib, an intracellular inhibitor of tyrosine kinases that has exhibited a satisfactory safety and tolerability record in pediatric patients [67].

In patients presenting with congenital pulmonary alveolar proteinosis (PAP) stemming from a granulocyte–macrophage colony-stimulating factor (GM-CSF) receptor mutation or an acquired receptor dysfunction due to autoantibody formation, a comprehensive assessment is warranted to determine the most suitable course of treatment. Subcutaneous or inhaled GM-CSF treatment has been reported that this is beneficial [68].

ABCA3 (adenosine triphosphate-binding cassette transporter A3) is a phospholipid transporter that is important for pulmonary surfactant storage and homeostasis. Cyclosporin A, a calcineurin inhibitor, was determined to be a correction for some, but not all, ABCA3 variants [69]. This gene is a member of the same gene family as the cystic fibrosis transmembrane conductance regulator (CFTR). Ivacaftor and genistein, as CFTR potentiators, have demonstrated the ability to enhance CFTR channel opening, resulting in various advantageous effects such as improved lung function, surfactant function, weight gain, and fertility. It may represent a therapeutic option for patients experiencing surfactant deficiencies attributed to *ABCA3* mutations [70]. In 2018, Kinting et al. demonstrated that the bithiazole correctors C13 and C17 effectively restored the functionality of all mutant proteins, apart from M760R ABCA3. Furthermore, these variants exhibited positive responses to the chemical chaperone trimethylamine N-oxide and low-temperature conditions. The discovery of the lead molecules C13 and C17 represents a significant milestone in the development of pharmacotherapy for ABCA3 misfolding-induced lung disease [71].

Mesenchymal stromal cells (MSCs) may be a potential therapy for incurable lung disease in both the adult and pediatric populations [72]. Stem cell therapy for ILD improves the quality of life through reducing the severity of symptoms, slowing disease progression, reducing lung inflammation, and significantly improving lung function. MSCs are commonly delivered via intratracheal or intravenous administration [73]. Induced pluripotent stem cells have been employed in a mouse model of pulmonary alveolar proteinosis (PAP) precipitated by CSF2RB deficiency [72,74,75].

Gene therapy could be a highly promising treatment option for monogenic lung diseases, which is now realizable [76,77,78]. The advancement of gene therapies for genetic disorders associated with surfactant dysfunction is intricately linked to the strategic design of viral vectors (retroviral vectors, lentiviral vectors, adeno-associated viral vectors, and adenovirus-based vectors) and their capacity to selectively target specific cell types. This process presents a spectrum of achievements and obstacles, underscoring the critical nature of viral vector development in the realm of gene therapy for surfactant dysfunction-related genetic disorders [79,80].

## 6. Limitations

This study aims to provide a comprehensive overview of interstitial lung disease in pediatric patients. However, some limitations should be acknowledged. Interstitial lung diseases are very rare in childhood and experience with these conditions is limited even in big pediatric respiratory centers. At present, there is no consensus on pediatric classification. Even though there has been significant progress, definitive therapies are often still not available. Current data regarding lung transplantation within the scope of medical interventions are limited.

## 7. Conclusions

Childhood interstitial lung disease (ILD) encompasses a wide array of rare and heterogeneous pulmonary disorders affecting the pediatric population. The clinical presentation of these diseases can manifest in a highly variable manner, necessitating consideration of a diverse range of potential symptoms and indicators for accurate diagnosis and management. It is essential to emphasize the pivotal role of chest imaging techniques, such as high-resolution computed tomography (HRCT), in the comprehensive evaluation and diagnostic process for childhood ILD.

One notable challenge in managing childhood ILD is the reliance on empirical pharmacological treatments, often without the support of controlled clinical trials. This underscores the imperative need for more rigorous and evidence-based approaches to medical interventions for these conditions. Furthermore, the scarcity of available data on lung transplantation in pediatric ILD patients necessitates a comprehensive understanding of the potential benefits and limitations of this therapeutic option in the management of severe and progressive cases.

Moreover, extensive clinical studies have provided compelling evidence to support the notion that ILD presents differently in children compared to adults. This highlights the substantial impact of additional factors related to lung growth and development on the natural history and disease progression of ILD in the pediatric population. A deeper understanding of these factors is crucial for tailoring effective treatment strategies and ensuring optimal outcomes for pediatric ILD patients [62,81].

We attempted to compose a detailed review article exploring the complexities of the subject and providing a thorough analysis. Our aim was not to perform a systematic review addressing a specific scientific question.

## Figures and Tables

**Table 1:** Categorization of chILD by etiology.

Known Etiology	Systemic Disorders with Pulmonary Involvement	Unknown Etiology
Chronic infections: viruses (EBV, CMV, adeno, hepatitis C, SARS-CoV-2, etc.), mycoplasma, chlamydia, mycobacteria, candida, pneumocystis, etc. Aspiration Hypersensitivity pneumonitis (drugs, environmental exposures) Bronchopulmonary dysplasia Genetic causes of surfactant deficiency (*SPFTB*, *SPFTC*, *ABCA3*, *SLC34A2* mutations) Desquamative interstitial pneumonitis (DIP) Pulmonary alveolar proteinosis (PAP) Pulmonary alveolar microlithiasis (PAM) Nonspecific interstitial pneumonitis (NSIP)	Connective tissue disorders (RA, SLE, dermatomyositis) Malignancies Langerhans cell histiocytosis Wegener granulomatosis Goodpasture disease Sarcoidosis Neurocutaneous syndromes (neurofibromatosis, tuberous sclerosis) Storage diseases (Gaucher, Niemann–Pick) Hermansky–Pudlak syndrome	Usual interstitial pneumonitis (UIP) Lymphocytic interstitial pneumonitis (LIP) Pulmonary infiltrates with eosinophilia (PIE) Pulmonary interstitial glycogenosis (PIG) Neuroendocrine cell hyperplasia in infancy (NEHI) Bronchiolitis obliterans Bronchiolitis obliterans with organizing pneumonia (BOOP) Idiopathic pulmonary hemosiderosis Pulmonary vascular disorders Pulmonary lymphatic disorders

**Table 2:** Classification proposed by ATS (chILD Research Network) (Kurland et al., 2013) [22].

**D** **isorders More Prevalent in Infancy**
A. Diffuse developmental disorders (e.g., congenital alveolar dysplasia)
B. Growth abnormalities (e.g., chronic neonatal lung disease)
C. Specific conditions of undefined etiology (e.g., pulmonary interstitial glycogenosis)
D. Surfactant dysfunction mutations and related disorders (e.g., *SPFTB* genetic mutations)
**Disorders not Specific to Infancy**
A. Disorders of normal hosts (e.g., infectious and postinfectious processes)
B. Disorders related to systemic disease processes (e.g., immune-related disorders)
C. Disorders of immunocompromised hosts (e.g., opportunistic infections)
D. Disorders masquerading as interstitial disease (e.g., arterial hypertensive vasculopathy)
Unclassified (e.g., end-stage disease)

**Table 3:** Differential diagnosis of interstitial lung disease in childhood by clinical presentation, radiology and histology findings, and possible genetic mutations [15].

Presentation		Radiology (CT)	Histology	Possible Genetic Mutations
**Neonatal period**	term neonates: unexplained respiratory distress (need for rapid intubation and ventilation) preterm neonates: respiratory distress more severe than expected due to prematurity	normal, ground-glass opacification, consolidations	pulmonary alveolar proteinosis, desquamative interstitial pneumonia, nonspecific interstitial pneumonia, chronic pneumonitis of infancy, alveolocapillary dysplasia with misalignment of pulmonary veins, alveolar simplification, acinar dysplasia, pulmonary interstitial glycogenosis	*ABCA3*, *SFTPB/C*, *NKX2-1*, *TBX4*, *FOXF1*, *TBX4*, *NKX2-1*
**First two years of life**	range from no symptoms to severe respiratory distress usually triggered by viral infections nonspecific signs and symptoms: tachypnea, dyspnea, failure to thrive, recurrent respiratory infections, exercise intolerance, dry cough, wheeze in the absence of respiratory tract infection, recession, pulmonary hypertension, gastroesophageal reflux, chest deformity	consolidations, air trapping, hyperinflation, ground-glass opacification, reticulation, cysts, periventricular heterotopia	pulmonary alveolar proteinosis, desquamative interstitial pneumonia, nonspecific interstitial pneumonia, chronic pneumonitis of infancy, alveolar simplification, infections	*NKX2-1*, *ABCA3*, *SFTPC*, *CSF2RA*, *CSF2RB*, *GATA2*, *MARS*, *OAS1*, *SLCA7*, *FLNA*
**Older children**	exercise-induced breathlessness, tachypnoea, hypoxia, cyanosis during exercise or at rest, digital clubbing auscultation is variable: no pathological sound, sometimes crackles coupled with wheezing	intra-alveolar calcification, ground-glass opacification, reticulation, cysts	microlithiasis, follicular bronchitis, pulmonary alveolar proteinosis, infections (CMV), pneumonitis, capillaritis, alveolar hemorrhage	*FLNA*, *COPA*, *STAT3*-GOF, *HPS1*,*3*,*4*,*5*,*6*, *AP3B1*, *DTNBP1*, *TMEM137*, *SCL34A2*, *SFTPC*

**Table 4:** Genetic mutations associated with children’s interstitial lung disease were proposed by Pelizzo in 2021 [38].

Genetic Mutation	Inheritance	Lung Involvement
*SFTPB* (surfactant protein B deficiency)	Autosomal recessive	Surfactant disorder
*SFTPC* (surfactant protein C mutation)	Autosomal dominant	Surfactant disorder
*CSF2RB* (colony-stimulating factor 2 receptor β)	Autosomal recessive	Pulmonary alveolar proteinosis
*CSF2RA* (colony-stimulating factor 2 receptor α)	X-linked	Pulmonary alveolar proteinosis
*ABCA3* (ATP-binding cassette-family A-member 3)	Autosomal recessive	Deficit surfactant
*COPA* (coatomer-associated protein subunit alpha)	Autosomal dominant	General disorder including lung
*FLNA* (Filamin A)	X-linked recessive	General disorder including lung
*FOXF1* (forkhead box F1)	Autosomal dominant	Alveolar capillary dysplasia
*GATA2* (GATA Binding Protein 2)	Autosomal dominant	Pulmonary alveolar proteinosis
MARS (metionil-transfer RNA sintetasi)	Autosomal recessive	Pulmonary alveolar proteinosis
*NKX2-1* (NK2 homeobox 1)	Autosomal dominant	Interstitial lung disease
*NSMCE3* (non-structural maintenance of chromosome element 3 homolog)	Autosomal recessive	Immunodeficiency
*OAS1* (oligoadenylate synthetase 1)	Autosomal dominant	Pulmonary alveolar proteinosis
*SLC7A7* (solute carrier family 7 member 7)	Autosomal recessive	Surfactant disorder
*TBX4* (T-box transcription factor 4)	Autosomal dominant	Acinar dysplasia
*TMEM173* (transmembrane protein 173)	Autosomal dominant	Lung fibrosis with general inflammation

**Table 5:** Proposed EU protocol (Delphi consensus according to Bush et al. (2015)) [15].

chILD (Ventilated or Close to Ventilation)		chILD (Not Ventilated or Close to Ventilation)
Methylprednisolone		
Dose	10 mg/kg or 500 mg/m^2^ (intravenous)	10 mg/kg or 500 mg/m^2^ (intravenous)
Response rate	7 days	28 days
Comment	A dose of 30 mg/kg is used by some centers.	An alternative to oral prednisolone. Use before other therapies and judge response.
Prednisolone		
Dose	Doses of 1 mg/kg (oral) are used between pulses of methylprednisolone.	Doses of 2 mg/kg (oral) are used as an alternative to methylprednisolone pulses. Use before other therapies and judge response.
Response rate	7 days	28 days
Hydroxychloroquine		
Dose	10 mg/kg	10 mg/kg
Response rate	21–28 days	3 months
Comment	In children <6 years, 6.5 mg/kg is used in some centers to reduce toxicity.	In children <6 years, 6.5 mg/kg is used in some centers to reduce toxicity. No preference over azithromycin as a second-line treatment. In addition, 54% would consider hydroxychloroquine as a sole therapy in mild stable chILD.
Azithromycin		
Dose	10 mg/kg 3 days per week	10 mg/kg 3 days per week
Response rate	3 months	3 months
Comment		No preference over hydroxychloroquine as a second-line treatment. In addition, 51% would consider azithromycin as a sole therapy in mild stable chILD.
